# Further Light on Irish Problems

**Published:** 1914-02-21

**Authors:** 


### FURTHER LIGHT ON IRISH PROBLEMS.

Familiar as Englishmen are with the intricate and far-reaching effects of the Insurance Act upon medical practice, they have little time to spare for consideration of the case of their Irish colleagues. Ireland possesses to all intents a separate Act, or, at least, a separate scheme of National Insurance totally different from that which is in operation in Great Britain. Some of the results upon the Irish medical profession were set forth in The Hospital last week. Other problems created by the Act are discussed in a leading article by the *Irish Times*. The system of certification set up by the Irish Insurance Commissioners is there described as a general failure. Members of the Irish Medical Committee openly announce the same view. One of them, Dr. M. R. J. Hayes, says that only a small minority of the Irish profession have consented to work it, and that it satisfies nobody. A demand is now being made for the appointment of a large number of whole-time medical officers for purposes of certification. This demand Dr. Hayes strenuously opposes. It will be apparent from this brief synopsis of current events that the Act is as great a source of heart-burning, intrigue, and dissatisfaction in Ireland as in the rest of the kingdom.

The Irish Medical Association and "The Lancet."

[In connection with a paragraph in last week's issue of The Hospital (p. 546), the Editor of the *Lancet* informs us that certain negotiations, marked "Confidential," were commenced by him and the Manager of the *Lancet* in reply to several requests from the Irish Medical Association. There was no suggestion that every member of the Irish Medical Association could have the *Lancet* supplied to him for 16s. from the office of the *Lancet*; the suggestion was that 600 copies were to be sent to Dublin for further distribution, and the £480 per annum in return was to be an inclusive subscription to the *Lancet*, open to a yearly revision of contract. When the question of an inset, apparently to be prepared by the Irish Medical Association, was made, the Manager wrote that such an inset could only appear at the discretion and under the control of the Editor of the *Lancet*, and no more was heard of the matter. While freely accepting this statement, it only remains to add that our paragraph was founded on a report of a meeting of the Council of the Irish Medical Association, published in that Association's official *Journal*. There was nothing in that report to indicate that confidential matters were being disclosed, and in any case it is obvious that any breach of confidence is not ours, but that of the newspaper from which our information was derived.—Ed. The Hospital.]

